# The Impact of the SARS-COV-2 Pandemic on the Mental Health and Employment Decisions of Medical Students in North China

**DOI:** 10.3389/fpsyt.2021.641138

**Published:** 2021-07-19

**Authors:** Feng Gao, Shu-xin Jiao, Ya-qiong Bi, Zi-yi Huang, Pei Wang, Bo-yan Zhang, Jing Fang, Rui-lan Han, Lei Fan, Min-jie Wang, Xiao-li Lv, Jun Li, Yu-xia Hu, Meng-di Zhang, Qing Qiao, Xue Zhao, Dan Li, Zhi-bin Xiao, Fu-hou Chang, Tu-ya Bai

**Affiliations:** ^1^Department of Clinical Pharmacy, Institute of Pharmacy, Inner Mongolia Medical University, Inner Mongolia Jinshan Development Area, Hohhot, China; ^2^Inner Mongolia Autonomous Region Engineering Research Center of New Pharmaceutical Screening, Inner Mongolia Jinshan Development Area, Hohhot, China; ^3^Transformation Innovation Platform for Clinical Pharmacy, Inner Mongolia Medical University, Inner Mongolia Jinshan Development Area, Hohhot, China; ^4^Institute of Pharmacy, Inner Mongolia Medical University, Inner Mongolia Jinshan Development Area, Hohhot, China; ^5^Inner Mongolia Autonomous Region Academy of Traditional Medicine, Hohhot, China; ^6^Department of Basic Medicine Institute of Pharmacy, Inner Mongolia Medical University, Inner Mongolia Jinshan Development Area, Hohhot, China; ^7^The Center for New Drug Safety Evaluation and Research of Inner Mongolia Medical University, Inner Mongolia Jinshan Development Area, Hohhot, China; ^8^Sales Department, Tianjin Tasly Pharmaceutical Commercial Co.Ltd., Tianjin, China

**Keywords:** SARS-CoV-2, anxiety, stress, medical career, academic burden

## Abstract

**Background:** The outbreak of severe respiratory syndrome coronavirus 2 (SARS-COV-2) has led to long periods of social isolation for individuals across the world. Although medical students generally have a high prevalence of mental health problems, they have received less attention than other groups concerning the impact of SARS-COV-2. Therefore, the present study investigated the mental health status, risk factors, and protective factors for mental health problems in medical students in North China during the SARS-COV-2 pandemic.

**Methods:** A WeChat-based survey, which included the Depression Anxiety Stress Scale-21 and measures of social demographics, was performed twice. Risk and protective factors were identified by binary logistic regression analysis.

**Results:** A total of 702 effective questionnaires were collected in two separate surveys. In total, 24.55% of medical students were suffering anxiety to different degrees of severity, 13.18% were suffering depression in the first survey, and 3.71% wanted to give up working in primary medical care during the SARS-COV-2 pandemic in the second survey. In contrast, during the SARS-COV-2 pandemic, a risk factor for anxiety and depression was gender which is male, while being knowledgeable about the SARS-COV-2 pandemic and having a lower academic burden were both protective factors.

**Conclusions:** Measures are required to prevent increases in mental health problems in medical students. Our findings suggest that increasing knowledge about the SARS-COV-2 pandemic and reducing academic burden in medical students is extremely important during the SARS-COV-2 pandemic.

## Introduction

Severe respiratory syndrome coronavirus 2 (SARS-COV-2) is a beta coronavirus that emerged abruptly in Wuhan, China in December 2019 (1). A total of 322,46,752 cumulative confirmed cases and 983,250 cumulative deaths were reported worldwide as of September 25, 2020 (2). To reduce the spread of SARS-COV-2, social isolation was performed in every country (3). However, long-term social isolation can cause a series of mental health problems including stress, anxiety, and depression (4).

Results of a meta-analysis indicate a higher global prevalence of anxiety in medical students compared to the age-matched general population (5). In addition, a recent survey demonstrated that medical students have a greater likelihood of having mental health problems during the SARS-COV-2 pandemic due to having a deeper understanding of viral pandemics (4). However, medical students are a major component of medical reserve forces and have to undertake the responsibility of ensuring people's health and safety (6). Therefore, it is important to identify the mental health status, risk factors, and protective factors for mental health problems in medical students during the SARS-COV-2 pandemic.

The SARS-COV-2 prevalence has led to many urgent medical problems, including shortages of medical supplies and staff, as well as poor medical conditions, and there remains a weak awareness of pandemic prevention (7). Even before the SARS-COV-2 pandemic, only 15.96% of medical graduates in China continued to pursue a medical career and up to 84% of young doctors chose to give up their career, although the national medical expenditure and proportion of elderly individuals are constantly increasing (8). Thus, we speculate that difficulties in the accessibility and affordability of medical care will be enduring. Surprisingly, it is still unknown whether the SARS-COV-2 pandemic may lead to severe mental health problems and further aggravate the reluctance of medical students to devote themselves to medical careers.

In this study, we used the Depression, Anxiety and Stress Scale-21 (DASS-21) to assess the severity of SARS-COV-2 pandemic induced anxiety, depression, and stress among medical students (9). We also used binary logistic regression analysis to predict the risk and protective factors for psychological stress in medical students and investigated whether the SARS-COV-2 pandemic is impairing the willingness of medical students to pursue medical careers.

## Materials and Methods

### Participants and Sampling

The participants were undergraduate, postgraduate, and doctoral medical students at medical colleges in Inner Mongolia, Tianjin, Hebei, Jinzhou, and other provinces in the north that were not seriously affected by the epidemic. Participants were invited to complete the survey using QQ, WeChat, and other social networking sites, and guidance was provided by Questionnaire Star (https://www.wjx.cn). Both surveys were completed anonymously. The purpose of the survey was explained to participants who provided informed consent before starting the questionnaire.

The survey was conducted twice. The first survey was conducted from June 23, 2020 to July 19, 2020 to investigate the mental health status of medical students in communities or towns during the lockdown. A total of 400 questionnaires were collected, and 13 invalid questionnaires were removed from analysis leaving 387 questionnaires with an effective rate of 96.75%. The second survey was conducted from October 9, 2020 to October 11, 2020. A total of 334 questionnaires were collected, of which 315 were analyzed, reflecting an effective rate of 94.31%. Questions on employment-related issues were added to the second survey.

### Measures

#### Social Demographic Questionnaire

The questionnaire obtained measures of sociodemographic information, such as age, gender, residential area, grade, whether an only child or not, and height and weight, which was used to calculate the body mass index (BMI) of each participant. Some factors, including gender, smoking status, SARS-COV-2 pandemic knowledge, academic burden, and time of sleep, were identified from the literature and applied in this survey to explore the related risk and protective factors for psychological problems in medical students.

#### Depression Anxiety and Stress Scale-21

The Depression Anxiety and Stress Scale-21 (DASS-21) was developed in 1995 by Lovibond and Lovibond (10), in which all dimensions of the original full-length scale remain unchanged but simplified test items are used to improve the efficiency in identifying and evaluating the corresponding symptoms of psychological disorders.

The DASS-21 consists of three subscales with a total of 21 items to examine the extent of an individual's depression, anxiety, and stress. The possible responses of “Did not apply to me at all,” “Applied to me to some degree or some of the time,” “Applied to me to a considerable degree or a good part of the time,” and “Applied to me very much or most of the time” were used by participants in response to each item according to their feelings over the past week. The sum of the seven items on each subscale was multiplied by 2 to obtain the subscale score, which ranged from 0 to 42. Higher scores represented higher levels of psychological distress.

The subscale scores were interpreted according to cut-offs. For depression, these were: normal (0–9), mild (10–13), moderate (14–20), severe (21–27), and extremely severe (>28). For anxiety, the scores were: normal (0–7), mild (8–9), moderate (10–14), severe (15–19), and extremely severe (>20). For stress, the scores were: normal (0–14), mild (15–18), moderate (19–25), severe (26–33), and extremely severe (>34).

## Statistical Analysis

All statistical analyses were performed using SPSS version 24.0. Descriptive statistics and frequency counts are presented (%). Mann–Whitney *U*-tests and Kruskal–Wallis tests determined whether there were significant differences in the number of psychological problems. Binary logistic regression analysis was used to define the risk and protective factors of mental health difficulties. A *P*-value of < 0.05 was considered statistically significant.

## Results

The demographic characteristics of the total of 702 participants across the two surveys are presented including the gender, region, grade, only child, BMI and smoking in Table 1. In the first survey, of the 387 effective questionnaires, the majority of participants were female (71.32%), living in urban areas (62.27%), undergraduates (93.02%), and not only children (60.98%). The proportions of participants with a BMI of low, normal, overweight, and obese were 22.74, 61.5, 9.56, and 6.2%, respectively. In total, there were 17 smokers (4.39%).

**Table 1:** Demographic characteristics of the participants across the two surveys.

**Total**	**First survey participants**	**Second survey participants**
	**(*n* = 387)**	**(*n* = 315)**
**Gender**		
Male	111 (28.68%)	90 (28.57%)
Female	276 (71.32%)	225 (71.43%)
**Region**		
Urban	241 (62.27%)	191 (60.63%)
Rural	146 (37.73%)	124 (39.37%)
**Grade**		
Undergraduate	360 (93.02%)	290 (92.06%)
Non-undergraduate	27 (6.98%)	25 (7.94%)
**Only child**		
No	236 (60.98%)	185 (58.73%)
Yes	151 (39.02%)	130 (41.27%)
**BMI**		
Low	88 (22.74%)	84 (26.67%)
Normal	238 (61.5%)	194 (61.59%)
Overweight	37 (9.56%)	32 (10.16%)
Obese	24 (6.2%)	5 (1.59%)
**Smoking**		
No	370 (95.61%)	308 (97.78%)
Yes	17 (4.39%)	7 (2.22%)

Compared to the first survey, there were the total of 315 effevtive participants in the second survey, and it had a higher proportion of females (71.43%), participants living in rural areas (39.37%), those who were non-undergraduates (7.94%), only children (41.27%), and smokers (97.78%). The proportions of participants with a BMI of low, normal, overweight, and obese were 26.67, 61.59, 10.16, and 1.59%, respectively.

All valid questionnaires in total 702 across the two surveys were analyzed to show the proportions for the different levels of severity for depression, anxiety, and stress as shown in Table 2. The symptoms of depression, anxiety, and stress were divided into five levels including normal, mild, moderate, severe, and extremely severe. The results indicated that most participants across both surveys were healthy in terms of the measurement of depression, anxiety, and stress. The proportion of participants who were considered healthy in terms of depression in the first survey was 86.82%. The proportion of participants in the first survey considered normal in terms of anxiety was 75.45%, and normal in terms of stress was 88.37%. The proportion of participants who were normal in terms of depression in the second survey was 79.05%. The proportion considered normal in terms of anxiety in the second survey was 66.03%, and normal in terms of stress was 87.62%. In the first survey, the proportion of individuals with unhealthy symptoms of depression was 13.18%, the proportion of individuals with unhealthy symptoms of anxiety was 24.55%, and the proportion of individuals with unhealthy symptoms of stress was 11.63%. In the second survey, the proportion of individuals with unhealthy symptoms of depression was 20.95%, the proportion of individuals with unhealthy symptoms of anxiety was 33.97%, and the proportion of individuals with unhealthy symptoms of stress was 12.38%.

**Table 2:** The proportion of different levels of severity for depression, anxiety, and stress symptoms across the two surveys.

**Symptom**	**Depression**		**Anxiety**		**Stress**	
**Variables**	**First survey**	**Second survey**	**First survey**	**Second survey**	**First survey**	**Second survey**
	***n* (%)**	***n* (%)**	***n* (%)**	***n* (%)**	***n* (%)**	***n* (%)**
Normal	336 (86.82%)	249 (79.05%)	292 (75.45%)	208 (66.03%)	342 (88.37%)	276 (87.62%)
Mild	33 (8.53%)	24 (7.62%)	21 (5.43%)	26 (8.25%)	25 (6.46%)	20 (6.35%)
Moderate	13 (3.36%)	33 (10.48%)	48 (4.13%)	57 (18.1%)	13 (3.36%)	13 (4.13%)
Severe	4 (1.03%)	6 (1.9%)	16 (4.13%)	8 (2.54%)	4 (1.03%)	3 (0.95%)
Extremely severe	1 (0.26%)	3 (0.95%)	10 (2.58%)	16 (5.08%)	3 (0.78%)	3 (0.95%)

The binary logistic regression analysis was used to analyze the risk and protective factors for depression, anxiety, and stress in the first survey. Gender, region, smoking, SARS-COV-2 pandemic knowledge, BMI, academic burden, length of time spent with family every week, sleep duration per day, and time of sleep were brought into our survey to study the influence factors of depression, anxiety, or stress. As shown in Table 3, gender, smoking, SARS-COV-2 pandemic knowledge, schoolwork burden, sleep duration per day, and time of sleep as controlling variables all had a significant effect on depression, anxiety, or stress (*P* < 0.05), excluding the influence of region, BMI to the depression, the influence of region, BMI and length of time spent with family every week to the anxiety, and the influence of region to the stress ((*P* > 0.05).

**Table 3:** Risk and protective factors for depression, anxiety, and stress identified through binary logistic regression analysis (first survey).

	**Depression**					**Anxiety**					**Stress**				
	**No**	**Yes**	**Adjusted OR (95% CI)**	***P*****-value**		**No**	**Yes**	**Adjusted OR (95% CI)**	***P*****-value**		**No**	**Yes**	**Adjusted OR (95% CI)**	***P*****-value**	
	***n* (%)**	***n* (%)**		**Category**	**Overall**	***n* (%)**	***n* (%)**		**Category**	**Overall**	***n* (%)**	***n* (%)**		**Category**	**Overall**
**Gender**															
Male	85 (76.58%)	26 (23.42%)	2.337 (1.129–4.836)	0.022	0.000	67 (60.36%)	44 (39.64%)	2.603 (1.463–4.634)	0.001	0.000	89 (80.18%)	22 (19.82%)	1.856 (0.85–4.052)	0.121	0.001
Female	251 (90.94%)	25 (9.06%)	1 (ref)	NA		225 (81.52%)	51 (18.48%)	1 (ref)	NA		253 (91.67%)	23 (8.33%)	1 (ref)	NA	
**Region**															
Urban	215 (89.21%)	26 (10.79%)	0.430 (0.198–0.935)	0.033	0.075	188 (78.01%)	53 (21.99%)	0.566 (0.317–1.011)	0.054	0.134	218 (90.46%)	23 (9.54%)	0.511 (0.232–1.128)	0.097	0.101
Rural	121 (82.88%)	25 (17.12%)	1 (ref)	NA		104 (71.23%)	42 (28.77%)	1 (ref)	NA		124 (84.93%)	22 (15.07%)	1 (ref)	NA	
**Smoker**															
No	325 (87.84%)	45 (12.16%)	0.257 (0.064–1.036)	0.056	0.006	285 (77.03%)	85 (22.97%)	0.249 (0.072–0.863)	0.028	0.001	333 (90%)	37 (10%)	0.125 (0.034–0.459)	0.002	0.000
Yes	11 (64.71%)	6 (35.29%)	1 (ref)	NA		7 (41.18%)	10 (58.82%)	1 (ref)	NA		9 (52.94%)	8 (47.06%)	1 (ref)	NA	
**SARS-COV-2 pandemic knowledge**															
Familiar	98 (91.59%)	9 (8.41%)	0.211 (0.051–0.874)	0.032	0.001	81 (75.7%)	26 (24.3%)	0.25 (0.079–0.793)	0.019	0.000	99 (92.52%)	8 (7.48%)	0.079 (0.018–0.347)	0.001	0.000
Roughly familiar	225 (86.87%)	34 (13.13%)	0.364 (0.112–1.182)	0.092		203 (78.38%)	56 (21.62%)	0.208 (0.072–0.599)	0.004		231 (89.19%)	28 (10.81%)	0.165 (0.051–0.535)	0.003	
Know nothing	13 (61.9%)	8 (38.1%)	1 (ref)	NA		8 (38.1%)	13 (61.9%)	1 (ref)	NA		12 (57.14%)	9 (42.86%)	1 (ref)	NA	
**BMI**															
Low	81 (89.01%)	10 (10.99%)	0.232 (0.051–1.063)	0.060	0.113	68 (74.73%)	23 (25.27%)	0.427 (0.13–1.4)	0.160	0.128	85 (93.41%)	6 (6.59%)	0.075 (0.016–0.342)	0.001	0.005
Normal	209 (86.72%)	32 (13.28%)	0.438 (0.115–1.671)	0.227		188 (78.01%)	53 (21.99%)	0.386 (0.13–1.142)	0.085		213 (88.38%)	28 (11.62%)	0.223 (0.067–0.739)	0.014	
Overweight	32 (91.43%)	3 (8.57%)	0.188 (0.03–1.174)	0.074		25 (71.43%)	10 (28.57%)	0.455 (0.121–1.71)	0.244		31 (88.57%)	4 (11.43%)	0.243 (0.05–1.173)	0.078	
Obese	14 (70%)	6 (30%)	1 (ref)	NA		11 (55%)	9 (45%)	1 (ref)	NA		13 (65%)	7 (35%)	1 (ref)	NA	
**Heavy academic burden**															
Never	79 (88.76%)	10 (11.24%)	0.262 (0.073–0.945)	0.041	0.004	74 (83.15%)	15 (16.85%)	0.218 (0.073–0.655)	0.007	0.012	79 (88.76%)	10 (11.24%)	0.189 (0.052–0.687)	0.011	0.003
Sometimes	185 (90.24%)	20 (9.76%)	0.285 (0.092–0.883)	0.030		154 (75.12%)	51 (24.88%)	0.488 (0.187–1.275)	0.143		187 (91.22%)	18 (8.78%)	0.176 (0.055–0.559)	0.003	
Often	54 (81.82%)	12 (18.18%)	0.704 (0.205–2.413)	0.577		50 (75.76%)	16 (24.24%)	0.412 (0.138–1.231)	0.112		58 (87.88%)	8 (12.12%)	0.309 (0.083–1.147)	0.079	
Always	18 (66.67%)	9 (33.33%)	1 (ref)	NA		14 (51.85%)	13 (48.15%)	1 (ref)	NA		18 (66.67%)	9 (33.33%)	1 (ref)	NA	
**Length of time spent with family every week**															
0–1 h	38 (71.7%)	15 (28.3%)	3.207 (0.996–10.331)	0.051	0.001	37 (69.81%)	16 (30.19%)	0.959 (0.37–2.484)	0.931	0.282	41 (77.36%)	12 (22.64%)	1.725 (0.494–6.025)	0.393	0.028
1–3 h	106 (84.13%)	20 (15.87%)	2.190 (0.861–5.569)	0.100		90 (71.43%)	36 (28.57%)	1.576 (0.794–3.127)	0.194		111 (88.1%)	15 (11.9%)	1.623 (0.596–4.415)	0.343	
3–6 h	71 (92.21%)	6 (7.79%)	1.016 (0.314–3.287)	0.979		60 (77.92%)	17 (22.08%)	1.188 (0.532–2.652)	0.675		68 (88.31%)	9 (11.69%)	1.615 (0.527–4.95)	0.402	
>6 h	121 (92.37%)	10 (7.63%)	1 (ref)	NA		105 (80.15%)	26 (19.85%)	1 (ref)	NA		122 (93.13%)	9 (6.87%)	1 (ref)	NA	
**Sleep duration per day**															
>10 h	22 (78.57%)	6 (21.43%)	0.556 (0.097–3.198)	0.511	0.017	18 (64.29%)	10 (35.71%)	0.592 (0.138–2.548)	0.482	0.045	24 (85.71%)	4 (14.29%)	0.146 (0.02–1.058)	0.057	0.020
8–10 h	103 (86.55%)	16 (13.45%)	0.687 (0.174–2.708)	0.592		93 (78.15%)	26 (21.85%)	0.506 (0.152–1.678)	0.265		106 (89.08%)	13 (10.92%)	0.565 (0.14–2.28)	0.422	
6–8 h	196 (89.91%)	22 (10.09%)	0.314 (0.086–1.148)	0.080		169 (77.52%)	49 (22.48%)	0.428 (0.137–1.34)	0.145		197 (90.37%)	21 (9.63%)	0.342 (0.093–1.259)	0.107	
<6 h	15 (68.18%)	7 (31.82%)	1 (ref)	NA		12 (54.55%)	10 (45.45%)	1 (ref)	NA		15 (68.18%)	7 (31.82%)	1 (ref)	NA	
**Time of sleep**															
Before 10:00 pm	19 (95%)	1 (5%)	0.097 (0.009–0.987)	0.049	0.000	18 (90%)	2 (10%)	0.071 (0.012–0.412)	0.003	0.000	19 (95%)	1 (5%)	0.111 (0.009–1.319)	0.082	0.003
10:00 pm−11:00 pm	73 (92.41%)	6 (7.59%)	0.188 (0.049–0.727)	0.015		69 (87.34%)	10 (12.66%)	0.142 (0.048–0.419)	0.000		75 (94.94%)	4 (5.06%)	0.268 (0.059–1.216)	0.088	
11:00 pm−12:00 pm	152 (91.02%)	15 (8.98%)	0.247 (0.079–0.774)	0.016		133 (79.64%)	34 (20.36%)	0.27 (0.111–0.657)	0.004		152 (91.02%)	15 (8.98%)	0.476 (0.142–1.595)	0.229	
12:00 pm−1:00 am	68 (79.07%)	18 (20.93%)	0.649 (0.207–2.034)	0.458		54 (62.79%)	32 (37.21%)	0.614 (0.244–1.547)	0.301		70 (81.4%)	16 (18.6%)	1.215 (0.357–4.134)	0.755	
After 1:00 pm	24 (68.57%)	11 (31.43%)	1 (ref)	NA		18 (51.43%)	17 (48.57%)	1 (ref)	NA		26 (74.29%)	9 (25.71%)	1 (ref)	NA	

*OR, odds ratio; CI, confidence interval*.

Length of time spent with family every week also had a significant effect on depression (*P* < 0.05), whereas region or BMI did not (*P* > 0.05). It is therefore shown that knowledge about the SARS-COV-2 pandemic was a protective factor against depression [Odds ratio (OR), 0.211, 95% confidence interval (CI), 0.051–0.874]. Compared with the medical students who always had a heavy academic burden, those who never (OR, 0.262, 95% CI, 0.073–0.945) or sometimes (OR, 0.285, 95% CI, 0.092–0.883) had a heavy academic burden were protected from depression. The earlier participants went to bed, compared to going to bed after 1:00 a.m., was also a protective factor against depression, such as going to bed before 10:00 p.m. (OR, 0.097, 95% CI, 0.009–0.987). However, being male was a risk factor for depression (OR, 2.337, 95% CI, 1.129–4.836).

Not smoking was a protective factor against anxiety (OR, 0.249, 95% CI, 0.072–0.863). Compared with the medical students who know nothing about the SARS-COV-2 pandemic, those who had knowledge (OR, 0.25, 95% CI, 0.079–0.793) or even rough knowledge (OR, 0.208, 95%CI, 0.072–0.599) about the SARS-COV-2 pandemic knowledge were protected from anxiety. Never having a heavy academic burden, as compared to always having a heavy academic burden, was a protective factor against anxiety (OR, 0.218, 95% CI, 0.073–0.655). Compared with going to bed after 1:00 a.m., going earlier was also a protective factor against anxiety, such as before 10:00 p.m. (OR, 0.071, 95% CI, 0.012–0.412). Males (OR, 2.603, 95% CI, 1.463–4.634) were associated with a higher risk of experiencing symptoms of anxiety compared with females.

There was a significant effect on stress according to BMI group (*P* < 0.05). Non-smoking was a protective factor against stress (OR, 0.125, 95% CI, 0.034–0.459). Compared with the medical students who know nothing about the SARS-COV-2 pandemic, those that had knowledge (OR, 0.079, 95% CI, 0.018–0.347) or even some knowledge (OR, 0.165, 95% CI, 0.051–0.535) about the SARS-COV-2 pandemic were protected from stress. Compared with having an obese BMI, those with low (OR, 0.075, 95% CI, 0.016–0.342) or normal (OR, 0.223, 95% CI, 0.067–0.739) BMI were protected from stress. Compared with the medical students who always had a heavy academic burden, those who never (OR, 0.189, 95% CI, 0.052–0.687) or sometimes (OR, 0.176, 95% CI, 0.055–0.559) were protected from stress.

Finally, the findings from the second survey indicated that the employment intentions of 266 medical students (84.44%) had not been affected by the pandemic. However, 49 medical students (15.56%) had changed their employment intentions due to the pandemic. There were 180 respondents (57.14%) who chose medical institutions and 100 medical students (31.75%) continued to pursue advanced studies. Of these, 83 medical students (26.35%) chose medical institutions after their advanced studies (Table 4).

**Table 4:** Issues related to the employment and employment intentions of medical students.

	***n* (%)**
**Job-seeking area**	
Hospitals in first-tier and second-tier cities	189 (60%)
Hospitals in or under third-tier cities	107 (33.97%)
Primary medical institutions (community health service stations or township hospitals)	19 (6.03%)
**Primary health care institutions**	
Yes	192 (60.95%)
No	123 (39.05%)
**Reasons for not working in primary medical institutions**	
Low wages	72 (22.86%)
Poor working conditions	47 (14.92%)
Lower comprehensive strength of hospitals	41 (13.02%)
Few opportunities for promotion	155 (49.21%)
**Reasons for working in primary medical institutions**	
Preferential policies of the state	86 (27.3%)
Few job opportunities	45 (14.29%)
Less stress	55 (17.46%)
Make greater contribution at home	90 (28.57%)
Greater opportunity for promotion	39 (12.38%)
**Understanding of preferential policies for medical students to work in primary medical institutions**	
Yes	77 (24.44%)
No	238 (75.56%)
**Reasons for giving up medical career**	
Higher educational requirements	59 (18.73%)
Fierce competition and situation	88 (27.94%)
Strong work intensity	34 (10.79%)
Physician-patient conflict	65 (20.63%)
Undertaking risk to own life through work	14 (4.44%)
Low interest	21 (6.67%)
Low wages	21 (6.67%)
No chance for promotion	13 (4.13%)
**Employment intentions**	
Medical institutions	180 (57.14%)
Civil service	21 (6.67%)
Private enterprise	7 (2.22%)
Self-employment	7 (2.22%)
Continue to pursue advanced studies	100 (31.75%)
Medical institutions	83 (26.35%)
Civil service	11 (3.49%)
Private enterprise	5 (1.59%)
Self-employment	1 (0.32%)
**Alterations in employment intentions**	
Yes	49 (15.56%)
Continue working in primary medical care	39 (12.38%)
Give up working in primary medical care	10 (3.17%)
No	266 (84.44%)

## Discussion

Previous studies have shown that college students require psychological interventions when confronted by a series of large-scale stressors (4). Similarly, a meta-analysis indicated that medical students had a higher prevalence of anxiety compared to the age-matched general population (5). The present results extended these findings to show that during the SARS-COV-2 pandemic, 13.18% of medical students were in a state of mild, moderate, severe, or extremely severe depression and 24.55% of medical students were suffering from some severity of anxiety, 11.63% of medical students were undergoing stress in the first survey, and 3.71% of medical students had chosen to give up their medical careers in the second survey. In addition, a relative insufficiency of knowledge regarding the SARS-COV-2 pandemic and reporting a heavy academic burden were associated with a greater risk of mental health problems in medical students. We implemented this survey because studies on the mental health of medical students and employment intentions during the SARS-COV-2 pandemic are lacking, and recent studies have suggested the SARS-COV-2 pandemic is a major cause of stress in University students.

Previous research found that during the SARS-COV-2 pandemic, the incidence of stress, depression, and anxiety was higher in medical students compared with other college students (4). Our results add to previous findings by showing that the SARS-COV-2 pandemic and heavy academic burden are related to an increase in mental health problems in medical students. Although medical students have a deeper understanding of the SARS-COV-2 pandemic compared with other students, medical students with insufficient knowledge about the SARS-COV-2 pandemic seem more likely to experience mental health problems. Meanwhile, time spent with family and living distance from parents have both been inversely associated with mental health problems during the SARS-COV-2 pandemic (11). Our research suggests that promoting the spread of knowledge about the SARS-COV-2 pandemic may be an effective route to ensuring relief from stress, depression, and anxiety.

A retrospective study indicated that female medical students have a higher prevalence of anxiety (38.0%, 95% CI, 27.6–49.5%) than male students (27.6%, 95% CI, 19.3–37.8%), but this difference was not statistically significant (5). In contrast to this previous study, we found that the prevalence of anxiety was higher in male students than female students. This unexpected result may be caused by the gender balance of the medical students in the first survey. When comparing the prevalence of anxiety in medical students in different areas, we found that compared to urban medical students, anxiety and depression were increased among medical students living in rural areas, although there was no statistically significant difference (4). This could be explained by imbalances between economy, culture, and education, and different publicity efforts relating to pandemic prevention knowledge between urban and rural areas (12, 13). In addition, we found that the prevalence of anxiety in early and long sleepers was relatively lower, which is also consistent with previous research (14).

Our research also found that a lower academic burden and greater familiarity with the SARS-COV-2 pandemic were common protective factors for depression, anxiety, and stress. Not smoking and maintaining a normal work-rest schedule were protective factors for anxiety and stress. BMI index was a unique protective factor for stress. The overgeneralization of fear of the unknown is a burden to daily life and characteristic of mental problems such as posttraumatic stress disorder and anxiety disorders (15, 16). Owing to the abrupt occurrence of the SARS-COV-2 pandemic, people had insufficient knowledge about it and this lack of knowledge became a major factor for stress. Compared to other college students, medical training is academically and emotionally grueling. Therefore, academic burden is another major factor for stress in medical students (17). The present research suggests that increasing knowledge about the SARS-COV-2 pandemic and reducing academic burden in medical students is extremely important during the SARS-COV-2 pandemic.

According to a study by Lien et al., China educated a total of 4.7 million medical graduates between 2005 and 2015, of whom only 750,000 medical students became doctors, with 84% changing professions. That is to say, only 26% of 25–34-year-old doctors remained, and approximately 500,000 rural doctor positions were vacated (8). Our research extends these results to indicate that 3.71% of medical students wanted to give up working in primary medical care during the SARS-COV-2 pandemic. Thus, difficulties in the accessibility and affordability of medical services may last for a long time. These combined research findings suggest that universities need to take measures to reduce the prevalence of anxiety and the potential trend of giving up working in primary medical care among medical students.

We used a simplified version of the DASS, in which all dimensions of the original scale remain unchanged but simplified test items are used to improve the efficiency in identifying and evaluating the corresponding symptoms of psychological disorders (9, 18). Many studies have shown that the DASS-21 has the same stable factor structure and the same excellent reliability and validity as the full version of the DASS, but the DASS-21 is more suitable for rapid screening in research and clinical practice (9), supporting the use of the measure in this study.

We note a potential limitation to the current study. First, all participants were medical students based in North China. Therefore, our results only represent the population of undergraduate, postgraduate, and doctoral medical students in north China during the SARS-COV-2 pandemic. Although the characteristics of these students are representative of medical students more generally, studies in other districts of China are needed (16). Second, the survey was performed via the internet. The average time of answering questions was 4 min, and some less time-used questionnaire that were completed <4 min may lead to data inaccuracies (19). Meanwhile, the self-reporting of the levels of depression, anxiety, and stress may not be aligned with assessments conducted by mental health professionals (20). Third, the medical students sampled during the two surveys were not the same respondents and so the data cannot reflect psychological changes in medical students during the SARS-COV-2 pandemic. Fourth, a verification design for the risk and protective factors was not included in the present study. Finally, owing to the limited understanding of the SARS-COV-2 pandemic impact on medical care and education, the employment decisions of medical students were not included when we first designed the study.

In conclusion, we show for the first time that the SARS-COV-2 pandemic is associated with the employment choices of medical students in North China. A relative familiarity about the SARS-COV-2 pandemic and lower burden of academic work are protective factors for anxiety in medical students. Given the possibility of the SARS-COV-2 pandemic impacting the mental health and employment choices of medical students, this research provides evidence to support universities promptly taking measures to prevent mental health problems among medical students.

## Data Availability Statement

The raw data supporting the conclusions of this article will be made available by the authors, without undue reservation.

## Ethics Statement

This study was approved by Inner Mongolia Medical University of Science and Technology medical ethics committee. All subjects were informed about the purpose of the study in accordance with Chinese legislation. Written informed consent for participation was not required for this study in accordance with the national legislation and the institutional requirements.

## Author Contributions

FG, S-xJ, and Y-qB were withers for present research. Z-yH, PW, B-yZ, JF, R-lH, and LF were response for Questionnaire design. M-jW, X-lL, JL, Y-xH, M-dZ, XZ, DL, and Z-bX were response for data analysis. QQ, F-hC, and T-yB were response for manuscript reversion. All authors contributed to the article and approved the submitted version.

## Conflict of Interest

QQ was employed by company Tianjin Tasly Pharmaceutical Commercial Co. The remaining authors declare that the research was conducted in the absence of any commercial or financial relationships that could be construed as a potential conflict of interest.
